# Keystone Veterinary Medical Association

**Published:** 1898-01

**Authors:** W. L. Rhoads

**Affiliations:** Secretary


### KEYSTONE VETERINARY MEDICAL ASSOCIATION.

THE December meeting was held Tuesday evening, the 13th inst., in room No. 1, northwest corner Broad and Filbert Streets. The following members of the profession were present: Drs. Charles Williams, J. D. Houldsworth, J. O. George, William P. Phipps, Benjamin Underhill, J. J. Maher, W. M. Drake, A. M. Lushington, W. Horace Hoskins, Leonard Pearson, W. L. Rhoads, Thomas B. Rayner, C. J. Marshall; also Messrs. Megary, Jones, and Blount, of Veterinary Department of the University of Pennsylvania.

During the committee reports, Dr. Hoskins as Chairman of the committee to take up the work of the Association in the interest of a better meat-inspection for Philadelphia, in conjunction with the Woman's Health Protective Association, spoke of the meeting recently held by that society in which they had appointed a committee of five to specially take up the work of meat-inspection for Philadelphia.

The application of A. N. Lushington, V.M.D., was now reported and considered by the Censors The President appointed Dr. C. J. Marshall to fill vacancy upon said board. The application being favorably recommended, Dr. Lushington was unanimously elected to active membership, and introduced.

Dr. S. J. J. Harger, the essayist of the evening, telegraphed that he could not be present, so the President now called for the reports of cases, which could not be made at the last meeting for want of time. In response, Dr. Hoskins reported the case of a dog having been under observation for four years; came to hospital in October with a discharge from nostrils, loss of appetite, distressed breathing, enlargement of glands—especially thyroid, thymus, and inguinal—which were as large as eggs, dropsical effusion along thorax. Temperature $104\frac{3}{5}°$. The autopsy showed enlarged glands, liver pale, pulp of spleen increased and liquefied, intestines anæmic. He had Dr. Formad examine some sections from glands, and from him received the following report of conditions found: Hypertrophy of lymphatic glands. Lymphoma often found with blood-diseases; a condition very rare, but few cases being on record.

Dr. Pearson then spoke of a cat having pernicious anæmia that proved to be leukæmia, and asked Dr. C. J. Marshall to tell what he knew of the case, he having treated it. The cat was first treated for skin-trouble, which at times seemed to respond to treatment, then became worse. The glands became swollen, were at times very large, when, on the next day, they might be normal. Examination of blood showed white blood-corpuscles much enlarged. The autopsy proved this case to be one of leukæmia, a pathological condition seldom found, and one of which but few similar are upon record.

Dr. Pearson now spoke of two outbreaks of anthrax in the State, and said both were due to the working of foreign dried hides, and said the State intended to vaccinate the cattle in certain sections next season should the loss from anthrax exceed 4 or 5 per cent., as this was the percentage of loss in vaccination.

After the report of several interesting cases, Dr. Hoskins asked that the views of all present might be freely given as to the best place for the meeting of the United States Veterinary Medical Association for 1898.

It was the opinion of those present, both visitors and members, that the greatest good could be realized by meeting in Boston.

Dr. Hoskins now spoke of the money needed for the work now being done in the interest of better meat- and milk-inspection for Philadelphia.

It was moved and carried that the committee already appointed be authorized to solicit subscriptions for this purpose.

Notice was then given of the midwinter examination to be held by the State Veterinary Examination Board. The meeting then adjourned until January 11, 1898.

Dr. W. L. Rhoads,
Secretary.

